# Dutch healthcare reform: did it result in better patient experiences in hospitals? a comparison of the consumer quality index over time

**DOI:** 10.1186/1472-6963-12-76

**Published:** 2012-03-25

**Authors:** David E Ikkersheim, Xander Koolman

**Affiliations:** 1 KPMG Plexus, Breukelen, The Netherlands; 2 FALW, VU University, Amsterdam, The Netherlands

## Abstract

**Background:**

In 2006, the Dutch hospital market was reformed to create a more efficient delivery system through managed competition. To allow competition on quality, patient experiences were measured using the Consumer Quality index (CQI). We study whether public reporting and competition had an effect on the CQI between 2006 and 2009.

**Methods:**

We analyzed 8,311 respondents covering 31 hospitals in 2006, 22,333 respondents covering 78 hospitals in 2007 and 24,246 respondents covering 94 hospitals in 2009. We describe CQI trends over the period 2006-2009. In addition we compare hospitals that varied in the level of competition they faced and hospitals that were forced to publish CQI results publicly and those that were not. We corrected for observable covariates between hospital respondents using a multi level linear regression. We used the Herfindahl Hirschman Index to indicate the level of competition.

**Results:**

Between 2006 and 2009 hospitals showed a CQI improvement of 0.034 (p < 0.05) to 0.060 (p < 0.01) points on a scale between one and four. Hospitals that were forced to publish their scores showed a further improvement of 0.027 (p < 0.01) to 0.030 (p < 0.05). Furthermore, hospitals that faced more competition from geographically close competitors showed a more pronounced improvement of CQI-scores 0.004 to 0.05 than other hospitals (p < 0.001).

**Conclusion:**

Our results show that patients reported improved experiences measured by the CQI between 2006 and 2009. CQI levels improve at a faster rate in areas with higher levels of competition. Hospitals confronted with forced public publication of their CQI responded by enhancing the experiences of their patients.

## Background

In the last two decades, several Western countries introduced some form of managed competition in their health care system [1,2]. Common goal of these reforms is creating a demand driven system that provides more patient centered care [3]. To achieve this goal the quality of health care providers needs to be assessed and publicly reported [4,5]. Patients and health plans may then use quality information to make informed choices between health care providers.

The public reporting of provider quality can stimulate quality improvement through informed patient choice, quality contracting of providers by health plans and/or by intrinsic motivation of health care providers [6]. Previous studies have shown that public reporting of quality information does stimulate hospitals to initiate quality improvement projects. It remains unclear if--and to what extent--the introduction of managed competition combined with public quality reporting may affect quality [7-9].

We set out to answer this question in the context of the Dutch 2006 Health Insurance Act (HIA) reform. It was enacted to stimulate managed competition in the hospital market. Before the reforms all hospitals were financed through 'input' reimbursement. Hence hospital budget did not directly depend on production, but rather on historic agreements with the insurer regarding their budget. The after reform characteristics of the Dutch health can be summarized as followed: [2,10]

• There is a mandatory basic health insurance for everyone, purchased through private insurers (both profit and not-for-profit are possible);

• There is annual consumer choice of insurer and insurer products and choice between in kind and restitution policy;

• Insurers are obliged to have a system of open enrollment and are compensated via a risk-equalization system;

• There is a mandatory deductible of € 150 and avoluntary deductible up to € 650 per person per year;

• Insurers are expected to contract selectively with competing health care providers;

• The government requires health care providers to release quality information.

To enable provider competition, products were defined in terms of Diagnosis Treatment Combinations (DTCs), which are to some extent comparable to diagnosis related groups [11]. A number of initiatives were aimed at making provider quality transparent. These include widespread measurement of medical quality indicators that aim to indicate outcome utility and the measurement of the Consumer Quality Index (CQI) that aims to measure process utility. It is partly based on the Consumer Assessment of Healthcare Providers and Systems [12]. The CQI therefore is a partial measure of healthcare quality based on more objective consumer experiences rather than a more subjective satisfaction [13].

From 2006 onwards health plans were increasingly stimulated to negotiate with hospitals on price, quantity and quality of care. While in 2006 negotiations were restricted to products accounting for 7% of the total hospital budget, this was increased to 34% in 2009, and is expected to further increase to 70% in the near future. Consequently, hospitals are increasingly exposed to competition [14]. However, the bargaining power of health plans remains limited due to a limited use of selective hospital contracting by health plans. In addition health plans are currently reluctant with proactive member channeling toward preferred hospitals [15]. While this may have reduced competition during 2006-2009, the threat of significant competition in the future may have sparked hospital policy changes.

We examined whether the patient experiences of hospital care improved in the period 2006-2009. In addition we investigated whether forced public reporting of hospitals and higher levels of competition--in line with previous studies and policy objectives--were associated with better patient experiences [7,9,16,17].

## Methods

### Available data

Hospital care experiences of patients were measured in 2006, 2007 and 2009. Each year the questionnaire was sent to a random sample of patients who stayed at the hospital during the respective year. In 2006 31 hospitals out of total 94 Dutch hospitals participated in the measurement. This number increased to 78 in 2007 and 93 in 2009 [13].

Under Dutch law no specific ethical approval was required for our research as the respondents were informed during filling in the questionnaire that their answers may be used for research purposes.

The CQI consisted of seven quality aspects (Physician communication, Nurses communication, Pain treatment, New medication communication, Accommodation, Discharge information and Nurse services) in 2006. In 2007 eight quality aspects were added and one was deleted (see Table 1). In 2009 one quality aspect was deleted (see Table 1). All quality aspects were rated based on multiple answers which were scored at a four point scale. This scale varies between one for the lowest possible score and four indicating the highest possible score [13].

**Table 1:** number of questions per quality aspect

*Quality aspects*	2006	2007	2009
Physician communication	4	4	4

Nurses communication	4	4	4

Pain treatment	3	3	3

New medication communication	5*	2	2

Accommodation	4*	5	5

Treatment explanation		3	3

Feeling safe		5	5

Respect for autonomy		7	7

Contradictive information		3	3

Discharge information	7*	8	8

Ward intake experience		4	4

Intake conversation*		15*	12*

Hospital accessibility		3	3

Nurse services*	3*		

Intervention planning*		3*	

* Quality aspect and/or measurement for particular year is excluded from secondary analyses

Not all quality aspects were suitable for our analysis. The scale of the quality aspect 'information after discharge' was adjusted in 2007 and therefore lacks comparability between the years 2006 and later years. In the quality aspects New medication communication, Accommodation and Intake conversation, questions were deleted after analyses showed they did not discriminate between respondents. Consequently trends over time may be biased and these quality aspects were (partly) excluded from further analyses (see Table 1) [13].

### Primary analyses and publication

Each year the results for the individual hospitals were case mix corrected using multilevel linear regression analyses (consumer experiences were nested within hospitals) [18]. Means with comparison intervals (1.39 standard error) were calculated per quality aspect and per hospital adjusted for age, education and reported physical health, sex and reported mental wellbeing [19]. Next, hospitals were divided in three groups and given a 'star rating': one star if their interval was completely below the average across all providers, two stars if their interval overlaps with the average across all providers and three stars if their interval was completely above the average across all providers, so that the public could easily interpret the results of the individual hospitals.

Depending on the dominant health plan in the catchment area of the hospital, 16 out of 31 hospitals were confronted with forced publication their 2006 results. This decision was made by the health plan without consultation of hospitals. In 2007 none of the results were publicly reported. All 2009 results will be published at the health care portal http://www.kiesbeter.nl and some results are already publicly reported at health plans' websites. All hospitals have received their CQI results for each of the years [20].

### Secondary statistical analyses

We combined the data of the three years and conducted a multilevel linear regression analyses per quality aspect for the relevant years (2006/2007/2009 or 2007/2009). In addition we ran multilevel linear regressions for the combined quality aspects 1-3 (2006/2007/2009) and 4-12 (2007/2009), with quality aspects nested in individual respondents. In the regressions we corrected for the relevant case mix variables: age, education and reported physical health, sex and reported mental wellbeing.

We added dummies for groups of hospitals with different participation patterns to correct for different CQI scores between these groups of hospitals. To assess if hospitals which were confronted with published CQI results by health plans in 2006 outperformed hospitals which were not, we included a dummy variable for those hospitals which we included in our multi level linear regression model. In order to identify hospitals that faced more competition from neighboring hospital we used the Herfindahl Hirschman Index (HHI) of hospitals. This HHI was used as an explanatory variable in an ordinary least square (OLS) regression, while correcting for time effects and case mix variables [21]. All analyses were conducted in Stata/IC version 11.0.

## Results

### Respondents

In 2006 8,311 respondents filled out the questionnaire. The number of respondents per hospital varied from 177 to 365 (mean = 268.10; SD = 34.06). In 2007, 22,333 respondents (response = 61%) filled out the questionnaire and the numbers per hospital varied from 190 to 358 (mean = 286.32; SD = 36.61). In 2009, 24,246 respondents filled out the questionnaire (response = 57%) and the numbers per hospitals varied from 128 to 458 (mean = 257.93; SD = 56.30). Table 2 shows the non-response analyses, in 2009 respondents were significantly younger than non-respondents.

**Table 2:** Non-response analyses

	Respondents	Non-respondents
2006 (n) (31 hospitals)	8,311	unknown

% Male 2006	unknown	unknown

Avg. age 2006	61	unknown

2007 (n) (78 hospitals)	22,333	14,190

% Male 2007	42%	42%

Avg. age 2007	61	63

2009 (n) (94 hospitals)	24,246	18,009

% Male 2009	43%	42%

Avg. age 2009	60*	72

*Significant at p < 0.05, **significant at p < 0.01, ***significant at p < 0.001

### Change in performance over time

Table 3 displays the uncorrected mean and standard deviation per included quality aspect per included year. Both means and standard deviation seem fairly constant throughout the period 2006-2009.

**Table 3:** mean and standard deviation per quality aspect in 2006, 2007 and 2009

*Quality aspects*	2006		2007		2009	
	**Mean**	**SD**	**Mean**	**SD**	**Mean**	**SD**

Physician communication	3.43	0.57	3.41	0.58	3.49	0.52

Nurses communication	3.39	0.64	3.45	0.64	3.53	0.55

Pain treatment	3.49	0.65	3.46	0.66	3.45	0.67

New medication communication	n/a		3.05	0.96	2.98	0.95

Accommodation	n/a		3.29	0.53	3.25	0.54

Treatment explanation	n/a		3.47	0.70	3.52	0.65

Feeling safe	n/a		3.35	0.63	3.43	0.59

Respect for autonomy	n/a		2.97	0.64	3.02	0.64

Contradictive information	n/a		3.45	0.96	3.43	1.00

Discharge information	n/a		3.20	0.88	3.15	0.88

Ward intake experience	n/a		3.88	0.43	3.87	0.40

Hospital accessibility	n/a		3.48	0.76	3.49	0.73

In Table 4 the result of the multi level linear regression per separate quality aspect is presented. For the first three quality aspects (Physician communication, Nurses communication, Pain treatment) the regression spans the years 2006, 2007 and 2009. The time effects of the years 2007 and 2009 for the first three quality aspects are mixed with significant positive and negative coefficients. The latter nine quality aspects (New medication communication, Accommodation, Treatment explanation, Feeling safe, Respect for autonomy, Contradictive information, Discharge information, Ward intake experience, Hospital accessibility) show more consistent results: seven quality aspects show significant improvement in 2009 compared to 2007.

**Table 4:** Multi level linear regression: coefficients and significance per quality aspect with relevant years 2006/2007/2009(#) or 2007/2009

Coefficients per quality aspect****	Year 2007 (compared to 2006)	Year 2009 (compared to 2007)	Publication of results
Physician communication # ****	-0.039 **	0.014	0.032*

Nurses communication#	0.049 **	0.112 ***	0.021

Pain treatment#	-0.050*	-0.062 **	0.033

New medication communication	n/a	0.024	0.086

Accommodation	n/a	0.090***	0.045**

Treatment explanation	n/a	0.069 ***	0.033

Feeling safe	n/a	0.220 ***	0.025

Respect for autonomy	n/a	0.131 ***	0.033

Contradictive information	n/a	0.056 ***	0.026

Discharge information	n/a	0.010	0.026

Ward intake experience	n/a	0.027 **	0.077

Hospital accessibility	n/a	0.026	0.016

*Significant at p < 0.05, **significant at p < 0.01, ***significant at p < 0.001 **** Given the contradiction of the direction and significance of the year coefficients for the aspect 'Physician communication' we conducted a joint significance likelihood test. This test showed that chi^2 ^> 0.000 meaning that the results presented in table are correct as presented

Publication of results consequently has a non-significant consistent positive relationship with higher performance for 10 quality aspects. The quality aspects show significant improvement 'Physician communication' and 'Accommodation' at the p < 0.05 and P < 0.01 level.

Table 5 shows that the combined analyses of quality aspects yields consistent results: in 2009 hospitals improved their performance with 0.034 (p < 0.05) to 0.060 (p < 0.01) points compared to 2006 and 2009. Publication of results is associated with an improved the performance of hospitals of 0.027 (p < 0.01) to 0.030 (p < 0.05) points.

**Table 5:** Multi level linear regression; coefficients and significance of combined quality aspects

Coefficient of combined quality aspects	Year 2007	Year 2009	Publication of results
Quality aspects 1-3**** years 2006/2007/2009	-0.011	0.034 *	0.030 *

Quality aspects 4-12 years 2007/2009	n/a	0.060***	0.027**

*Significant at p < 0.05, **significant at p < 0.01, ***significant at p < 0.001 **** Given the contradiction of the direction and significance of the year coefficients for the aspect 'Physician communication' we conducted a joint significance likelihood test. This test showed that chi^2 ^> 0.000 meaning that the results presented in table are correct as presented

### Performance and level of competition

The aggregated analyses of quality aspects 1-3 and 4-12 shows that the HHI is inversely significantly related to the performance of hospitals at the p < 0.001 level, meaning that hospitals who face a higher level of competition outperform hospitals who face a lower level of competition, as shown in Table 6.

**Table 6:** OLS with HHI per quality aspect with relevant years 2006/2007/2009(#) or 2007/2009

Coefficient of combined quality aspects	HHI
Quality aspects 1-3# ****	-5.42 10^-6 ^***

Quality aspects 4-12	-4.89 10^-6 ^***

*Significant at p < 0.05, **significant at p < 0.01, ***significant at p < 0.001 **** Given the skewed distribution of the error term we estimated confidence intervals we applied a non parametric bootstrap procedure with 10,000 replications and hospital stratified sampling. Confidence intervals were based on bias corrected percentile score and the reported coefficients are within these confidence intervals, meaning that the found coefficients are significant. For additional analysis regarding potential outliers that may have influenced our regression results, please see Additional file 1. This analysis shows that this is not the case.

The coefficient is small, but this is largely caused by the scale of the HHI, which theoretically ranges from 1 to 10,000, but in our dataset ranges from 807 to 9,133. If one multiplies coefficients by these differences, the potential impact of the HHI is equal to a coefficient of approximately 0.004 to 0.05 points on the CQI which varies in our dataset between 2.97 and 3.88. Thereby this effect is in the same order of magnitude as the found time effects in Table 5.

## Discussion

We have examined the effects of the introduced transparency and competition between providers on patient experiences within the Dutch hospital market. This study is the first which systematically evaluates these effects within the Dutch setting. In addition we were able to use a dataset which enabled us to compare hospitals which were confronted with transparency early on with hospitals which were not. Our findings are as follows: First, we observe that patient experiences measured with the CQI improve over the course of years. Secondly, hospitals which are confronted with forced transparency early on are improving faster than hospitals which were not. This is evidence that the introduced transparency under the HIA may have improved quality of care, especially because patients' trust in hospitals shows the opposite trend, this declined over the same period from 68% to 66% [22]. Thirdly, we find that higher levels of competition adjusted for case mix differences and time effects are related with better patient experiences,.

Although the findings in our study are in line with previous studies our study may have suffered from possible limitations [8,23]. First, in this study we show that Dutch hospitals are improving their performance, but they found improvements are fairly small. It is well documented that CQI differences between good and bad performing providers are in the same orders of magnitude as we have found [24,25]. Therefore we believe that the differences in patient experiences between providers are meaningful and relevant for policy makers, health plans and hospitals. Moreover, we believe that current levels of competition are weak and our effects are likely to become stronger as competition increases. Secondly, and in line with our hypotheses and previous studies, our study indicates that a higher level of competition is related to better performance of hospitals. The effect of a higher level of competition on patient experiences is in the same order of magnitude as the found time effects [8,16]. We use the HHI to indicate the level of competition that is calculated in 2004. Although some mergers took place within the Dutch hospital market since 2004, changes in market share over time are fairly modest [26]. In addition, most mergers took place on a board level, while merging the enterprises of the medical specialists on the multiple locations often lags several years behind. This maintains the same pre-merger level of competition between medical specialists. Therefore we believe that adjusting the HHI over the course of years would not have altered our conclusions.

## Conclusions

In conclusion, our results show that patients reported improved experiences measured by the CQI between 2006 and 2009. CQI levels improve at a faster rate in areas with higher levels of competition. Hospitals confronted with forced public publication of their CQI responded by enhancing the experiences of their patients.

## Competing interests

The authors declare that they have no competing interests.

## Authors' contributions

DI drafted the manuscript and contributed to all other quality aspects of the study. XK was involved in the data analyses and performed critical revision of the manuscript. All authors read and approved the final manuscript.

## Pre-publication history

The pre-publication history for this paper can be accessed here:

http://www.biomedcentral.com/1472-6963/12/76/prepub

## Supplementary Material

Additional file 1 **Supplementary data**. Click here for file
