# Supplementary material for: Dutch healthcare reform: did it result in better patient experiences in hospitals? a comparison of the consumer quality index over time
Source: BMC Health Serv Res. 2012 Mar 25;12:76. doi: 10.1186/1472-6963-12-76 (PMC3326705; doi:10.1186/1472-6963-12-76)
Supplement: Additional file 1 — Supplementary data. [file 1472-6963-12-76-S1.DOC]

**Supplementary data**

In this supplementary data section we provide additional information regarding possible outliers that may have influenced our HHI regression, although the use of a non parametric bootstrap procedure would have resulted in large confidence intervals if outliers were of great influence.

In the study we estimate two regressions with the HHI, for both the aspects 1-3 and the aspects 4-12. Therefore we present the graphs separately. As one can see the regression of the HHI for the aspects 1-3 is not influenced by outliers in the figure S1 below.

Figure S1: Aspects 1-3 (x-axis: HHI of hospital and y-axis: average aspect score per hospital for aspects 1-3)

As one can see, the fitted line in the figure S2 below for the aspects 4-12 may have been influenced by the outlier hospital with an HHI around 7100. Therefore we ran an additional regression, excluding all hospitals with an HHI above 7000.

Figure S2: Aspects 4-12 (x-axis: HHI of hospital and y-axis: average aspect score per hospital for aspects 4-12)

The figure S3 below shows, that also after excluding all hospitals with a HHI above 7000, the slope of the line stays similar. Therefore we are confident that our results regarding the HHI regression are not influenced by outliers.

Figure S3: Aspects 4-12 (x-axis: HHI of hospital and y-axis: average aspect score per hospital for aspects 4-12, excluding hospitals with HHI higher than 7000)
